# Physical Body Orientation Impacts Virtual Navigation Experience and Performance

**DOI:** 10.1523/ENEURO.0218-23.2023

**Published:** 2023-11-23

**Authors:** Hyuk-June Moon, Hsin-Ping Wu, Emanuela De Falco, Olaf Blanke

**Affiliations:** 1 Neuro-X Institute, Faculty of Life Sciences, Swiss Federal Institute of Technology (EPFL), CH-1202 Geneva, Switzerland; 2 Brain Mind Institute, Faculty of Life Sciences, Swiss Federal Institute of Technology (EPFL), 1015 Lausanne, Switzerland; 3 Bionics Research Center, Biomedical Research Division, Korea Institute of Science and Technology (KIST), Seoul 02792, South Korea; 4 Department of Clinical Neurosciences, University Hospital Geneva, 1211 Geneva, Switzerland

**Keywords:** body orientation, MRI, navigation, position, VR

## Abstract

Most human navigation studies in MRI rely on virtual navigation. However, the necessary supine position in MRI makes it fundamentally different from daily ecological navigation. Nonetheless, until now, no study has assessed whether differences in physical body orientation (BO) affect participants’ experienced BO during virtual navigation. Here, combining an immersive virtual reality navigation task with subjective BO measures and implicit behavioral measures, we demonstrate that physical BO (either standing or supine) modulates experienced BO. Also, we show that standing upright BO is preferred during spatial navigation: participants were more likely to experience a standing BO and were better at spatial navigation when standing upright. Importantly, we report that showing a supine virtual agent reduces the conflict between the preferred BO and physical supine BO. Our study provides critical, but missing, information regarding experienced BO during virtual navigation, which should be considered cautiously when designing navigation studies, especially in MRI.

## Visual Abstract

**Figure:**
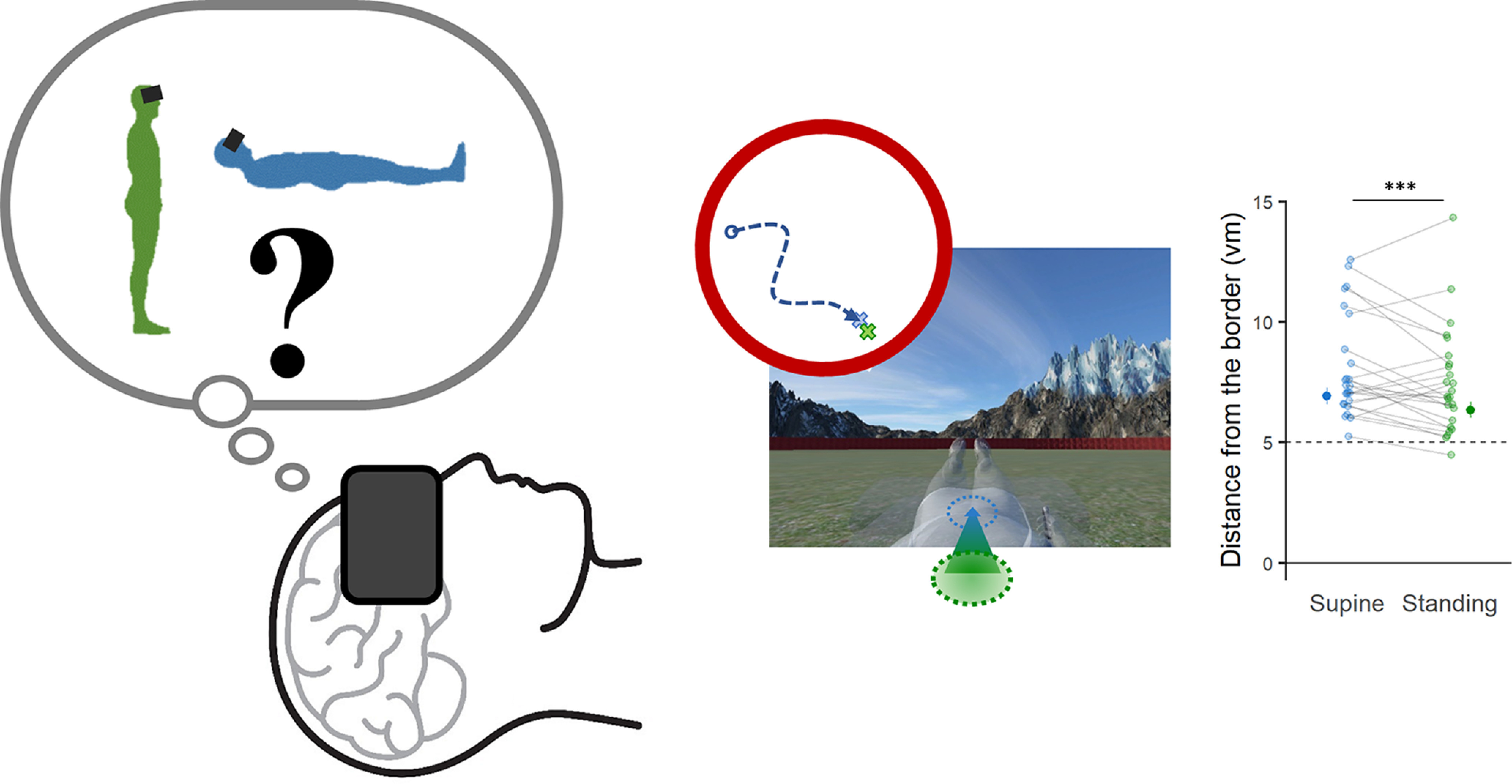

## Significance Statement

While virtual navigation studies in MRI have greatly contributed to our understanding of human spatial navigation systems, they have relied on a highly untypical navigation body orientation (BO) and experience: navigating while in a supine position. Whether such navigation BO and related experience influence navigation behavior is currently unknown. Investigating participants’ subjective reports and implicit navigational measures in supine and standing BO, we show that real-world BO influences BO experienced in VR, and it causes a conflict with preferred navigation BO (i.e., standing) when physically supine. Our results underline the importance of carefully considering the body and its orientation when designing virtual navigation studies.

## Introduction

The brain mechanisms of spatial navigation in humans are a prominent topic in the basic neurosciences (Maguire et al., 1999; Buzsáki and Moser, 2013; Ekstrom and Isham, 2017; Bellmund et al., 2018) and are of clinical relevance (delpolyi et al., 2007; Coughlan et al., 2018). The vast majority of human spatial navigation studies have used virtual navigation paradigms because of the fact that most of the noninvasive brain imaging techniques do not allow subjects to navigate in the real world and require participants to remain immobile. One of the most frequently used brain imaging techniques for human spatial navigation research is functional magnetic resonance imaging (fMRI). While fMRI provides access to neural activities in deep brain structures, including the medial temporal lobe (MTL), that are known to be crucial for spatial navigation (Scoville and Milner, 1957; Byrne et al., 2007; Moser et al., 2008; Squire, 2009), it requires participants not only to remain as immobile as possible, but also to be in a supine position. Although previous virtual navigation in fMRI significantly contributed to our understanding of human spatial navigation systems, the supine position in the MRI scanner imposes fundamental differences between virtual navigation “in the scanner” and ecological daily navigation “in the real world.” However, still, how these differences impact human navigation systems still needs further investigation (Taube et al., 2013; Park et al., 2018; Steel et al., 2021).

**Figure 1.**
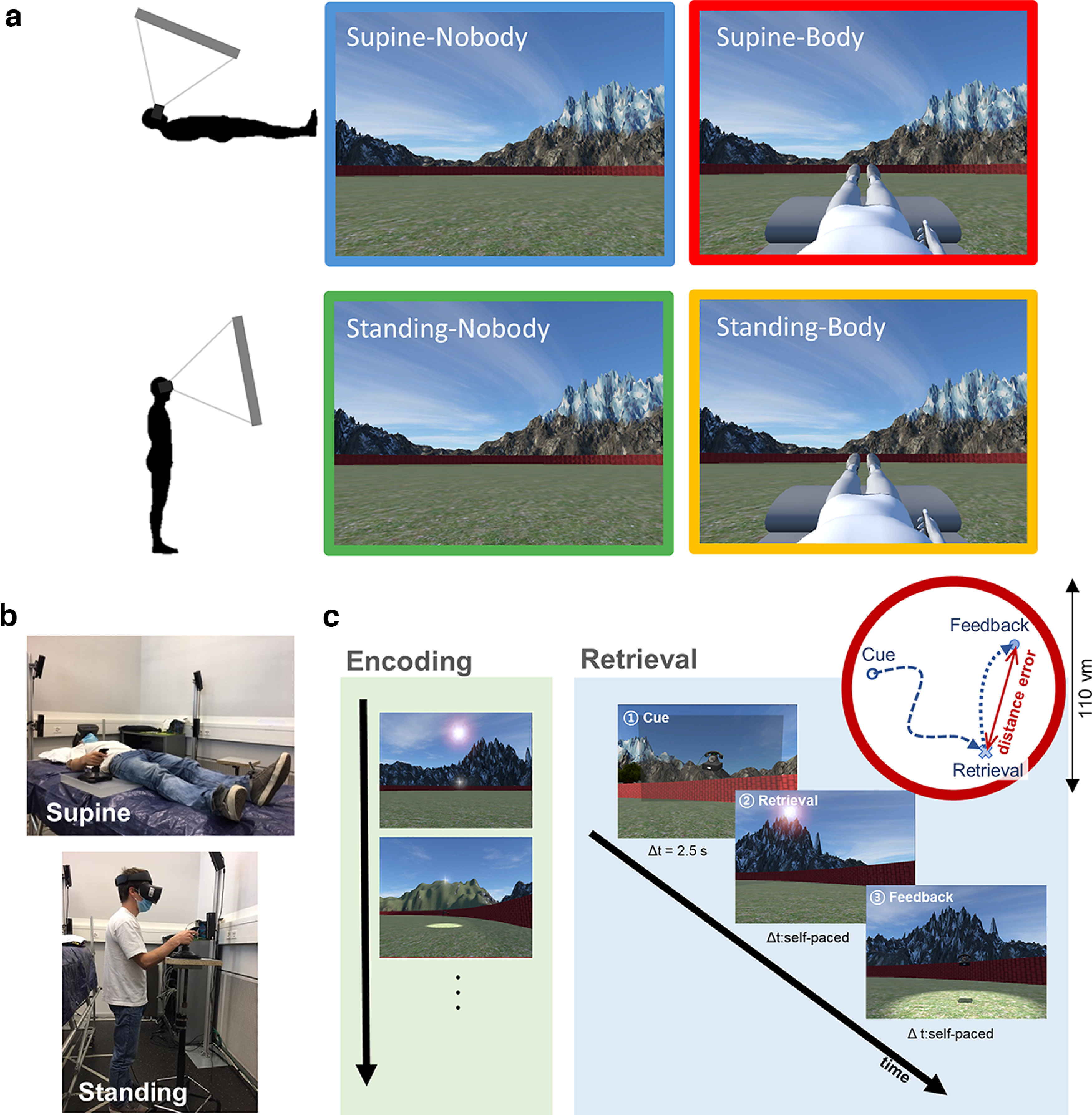
Experimental design combining two physical BOs and the presence/absence of an avatar conditions. ***a***, Participants performed the task in four different conditions obtained by the combination of the physical BO (i.e., Supine vs Standing) and the presence/absence of the avatar (i.e., Body vs Nobody). Note that the avatar (when shown) was always in a supine position. ***b***, Participants performed the virtual navigation task, wearing a VR head-mounted display while either standing upright or lying supine on a bed. ***c***, During the task, participants were navigating in a circular virtual arena, performing a spatial memory retrieval. In Encoding session, preceding each Retrieval session, a participant memorized locations of the target objects. At each trial of Retrieval session: (1) the image of the target object was shown; (2) a participant navigated to the retrieved location and responded; and (3) as feedback, the target object appeared at its correct location to be collected, vm: virtual meter.

Thus, it is unknown whether and how differences in physical body orientation (BO) affect (1) subjective experience of BO during virtual navigation (i.e., what is the experienced BO during virtual navigation when subjects are in a supine physical BO?) and (2) spatial navigation performance (e.g., navigation accuracy or speed). To the best of our knowledge, no study has addressed this issue directly. Also, it is often assumed that, regardless of their physical BO, participants in navigation studies experience themselves as if they were standing upright during virtual navigation (Jacobs et al., 2013; Taube et al., 2013; Maidenbaum et al., 2018). However, as suggested by Moon et al. (2022), bodily signals (e.g., vestibular and proprioceptive signals) from the physical body (i.e., supine participants in the scanner) can affect how participants experience the virtual agent in a virtual reality (VR) environment. The bodily reference frame of the participant’s physical BO may thus be in conflict with the bodily reference frame of the virtual agent’s BO (and such a mechanism may even be at play when no navigating avatar is shown in the virtual environment, as done in most previous spatial navigation research). In the present study, we hypothesized that the subjectively experienced BO in the virtual navigation space (as well as spatial navigation behavior) depends on the participant’s physical BO (in the scanner; mediated through intrinsic bodily signals) and, additionally, on whether an avatar is shown in VR or not. To test this hypothesis, we asked participants to perform the same, classical, spatial navigation task (Doeller et al., 2010) in two different physical BOs (either supine or standing). During the task, participants had to recall the location of a cued object that they had previously encoded and to navigate to the retrieved location. Based on findings by Moon et al. (2022), we also tested two additional conditions where a supine virtual agent was shown or not (Fig. 1*a*; Materials and Methods). We tested within-subject effects, and, thus, all participants went through the four experimental conditions.

## Materials and Methods

### Participants

Twenty-five healthy participants (11 males and 14 females; mean age, 25.7 ± 1.91 years) participated in the study. They gave informed consent following the institutional guidelines and the Declaration of Helsinki (2013). They were neither aware of the purpose of the study nor had a history of psychological disorders. They were right handed with normal or corrected-to-normal vision. They were recruited from the general population through the online recruitment system and received monetary compensation according to the contributed time [20 CHF (Swiss Francs)/h]. The number of participants (25) was chosen based on a power analysis conducted in a previous study that used a similar spatial navigation paradigm (Moon et al., 2022). Power analysis was performed using “simr” in the R package (version 1.0.6) on the previous dataset and concluded that a sample size of 25 was sufficient to reproduce the border effect between Body and Nobody conditions with a power of 88.5%. Participants who quit the experiment (four; three females) because of severe motion sickness were already excluded from the dataset and are not counted in the sample size.

### VR spatial navigation task with head-mounted display

The spatial navigation task used in the study was implemented with Unity Engine (Unity Technologies) by adapting the paradigm from the previous study (Moon et al., 2022). The participants wore the head-mounted display (HMD; Oculus Rift S, Oculus) and used a gaming joystick (Extreme 3D Pro, Logitech) to perform the task in the circular virtual arena. Distal landmarks were placed outside of the task arena, providing orientation cues to the participants. We disabled head tracking of the HMD system during the task, so that our participants saw the equivalent scene from a fixed angle of 15° regardless of the experimental conditions (Fig. 1*a*). This was (1) to control visual input and only investigate the impact of physical BO during virtual navigation and (2) to replicate the typical virtual navigation environment used in MRI studies.

Each session of the task began with an encoding phase, during which participants navigated the arena and encoded the positions of the three task objects placed within it. Next, they performed the recall task composed of 14 trials. In each recall trial, the target object was shown for 2 s (i.e., Cue phase) and participants had to recall its original location and navigate to that location (i.e., Retrieval phase). Upon the retrieval response, distance error was calculated as the Euclidean distance between the retrieved location and the correct location of the target object. Distance error inversely indexes spatial navigation accuracy (Fig. 1*c*; i.e., the larger the error, the lower the accuracy). In addition, navigated distance and time to retrieve were also recorded as additional measures of navigation performance. After the response, the object reappeared in its correct position, providing feedback to the participants, and had to be collected again to trigger the start of the next trial. In the last trial of each round, a threatening scenario (i.e., a virtual knife approaching from the sky toward either the avatar or the space where the avatar should have been placed in the Nobody condition) was presented to the participants. This was to provide an additional measure (i.e., response to the threat) of self-identification and self-projection of participants, the association between the virtual agent in VR and the sense of self. The last trials (with a threatening scenario) were excluded from the behavioral analyses.

The navigation task was performed in four different conditions obtained from the combination of the presence/absence of the avatar (i.e., Body vs Nobody) and the physical BO during the task (i.e., Supine vs Standing BO; Fig. 1). Of note, while the two manipulations generate four conditions, our design is not a two-by-two design. In fact, the avatar was only ever presented in a supine position (congruent to the Supine-Body condition), while no standing avatar was shown in the Nobody conditions. Therefore, we had no congruent avatar condition for the standing BO. The Supine-Body condition was adopted to assess the effect of a body-congruent avatar, as did Moon et al. (2022), while the Standing-Body condition was adopted to assess how incongruency between the participant’s physical BO and the BO of the avatar modulates subjectively experienced BO and spatial navigation performance.

The whole experiment was separated into two blocks. In each block, participants went through all four conditions presented in different orders. To avoid fatigue because of prolonged standing, physical BOs (i.e., standing and supine) were interleaved with each other within a block. The order of the conditions was pseudorandomized and counterbalanced between participants. In total, each participant performed eight sessions of the navigation task (twice per condition) and answered the questionnaires at the end of each session (see Questionnaire section for the details).

### Physical BO during the task

To assess the impact of the physical BO on the experienced BO during the virtual navigation, we asked participants to perform the experiment in two different physical BOs: in the supine BO condition, participants lay down on the bed with the joystick positioned on their right-hand side (Fig. 1*b*, left); in the standing BO condition (Fig. 1*b*, right), they stood upright and the joystick was positioned on a height-adjustable table on their right side. The height of the bed during the supine condition was set to approximately match the height of the participant’s upper trunk when they stood up.

### Virtual avatar during the task

A virtual avatar in a supine position was presented in the Body condition of the task (note that the avatar posture was always supine regardless of the BO condition). The movements of the right hand of the avatar were programmed to match the participant’s hand movements while controlling the joystick, providing a visuomotor congruency. In the supine BO condition with the avatar (i.e., Supine-Body condition), such visuomotor congruency, together with the visuoproprioceptive congruency of the physical BO and the BO of the virtual avatar, was expected to induce a higher illusory self-identification with the avatar during navigation. By contrast, in the standing BO condition with the avatar (i.e., Standing-Body condition), this visuoproprioceptive congruency was not met as the physical BO was incongruent with the BO of the avatar. Therefore, we expected lower self-identification with the avatar under this condition (Sanchez-Vives et al., 2010; Slater et al., 2010; Walsh et al., 2011; Kokkinara and Slater, 2014; Blanke et al., 2015).

### Questionnaire

At the end of each session, participants were asked to rate their agreement with seven statements (Q1 to Q7) using a Likert scale ranging from 0 (strongly disagree) to 6 (strongly agree). All the items are listed in Table 1. The order of the statements was shuffled at every session, and participants rated them autonomously with the joystick. Q1, Q2, and Q3 were aimed at assessing the bodily self-consciousness of the participants. Q6 and Q7 were designed to probe their experienced BO during the virtual navigation. The conflict between the physical BO and experienced BO was quantified as the conflict score, which was calculated with the participants’ physical BOs and the ratings of Q6 and Q7 by the following formula:

(1) Conflict Score = Q_Rating[incongruent BO] + (6 – Q_Rating[congruent BO])/2.

**Table 1:** Questionnaire items

Questions	Term	Description
Q1	Self-identification	I felt as if what I saw in the middle of the scene was my body
Q2	Threat	I felt as if the threat (knife) was toward me
Q3	Presence	I felt as if I was located in the virtual environment
Q4	Cyber-sickness	I felt dizzy
Q5	Control	I felt as if I had 3 bodies
Q6	Supine	I felt as if I was supine in the virtual environment
Q7	Standing	I felt as if I was standing in the virtual environment

At the end of each session, participants were provided seven questionnaire items and answered on a Likert scale ranging from 0 (strongly disagree) to 6 (strongly agree).

For instance, when a participant was physically supine, the conflict score was calculated as (Q_Standing + (6 – Q_Supine))/2: the higher when their experienced BO was opposed to the physical BO. In the formula, “6” stands for the maximum Q rating for the other questionnaire items as the conflict score was designed to be in the 0–6 range like the other questionnaire ratings. Q4 was aimed to capture the cyber-sickness during the task, while Q5 served as a general control question. We shortly debriefed the participant once the entire experiment was completed to ensure the integrity of their autonomous responses and also to record their spontaneous subjective reports.

### Statistical analysis

All the behavioral and questionnaire data were analyzed using R (version 4.1.2 for Windows; https://www.r-project.org/) and RStudio (version 2021.09.01; http://www.rstudio.com). The differences in the questionnaire ratings and conflict scores between conditions were assessed with a paired two-tailed Wilcoxon signed-rank test. For the behavioral parameters recorded at each trial (i.e., distance errors, navigation trace length and time, distance from the border), we performed mixed-effects regressions (lme4, version 1.1–18-1) with a fixed effect of condition and random intercepts for individual participants to assess statistical significance. Random slopes were assumed as far as the model did not fail to converge. We also examined the correlations between parameters using mixed-effect regression models. The distribution of each dependent variable was considered in the mixed-effect modeling of the variable, following the previous study using a similar task (Moon et al., 2022).

### Data availability

The data that support the findings of this study and the analysis code are available in the public repository (https://osf.io/rz8eg).

## Results

### Participant’s physical BO significantly affects the experienced BO and navigation behavior

**Figure 2.**
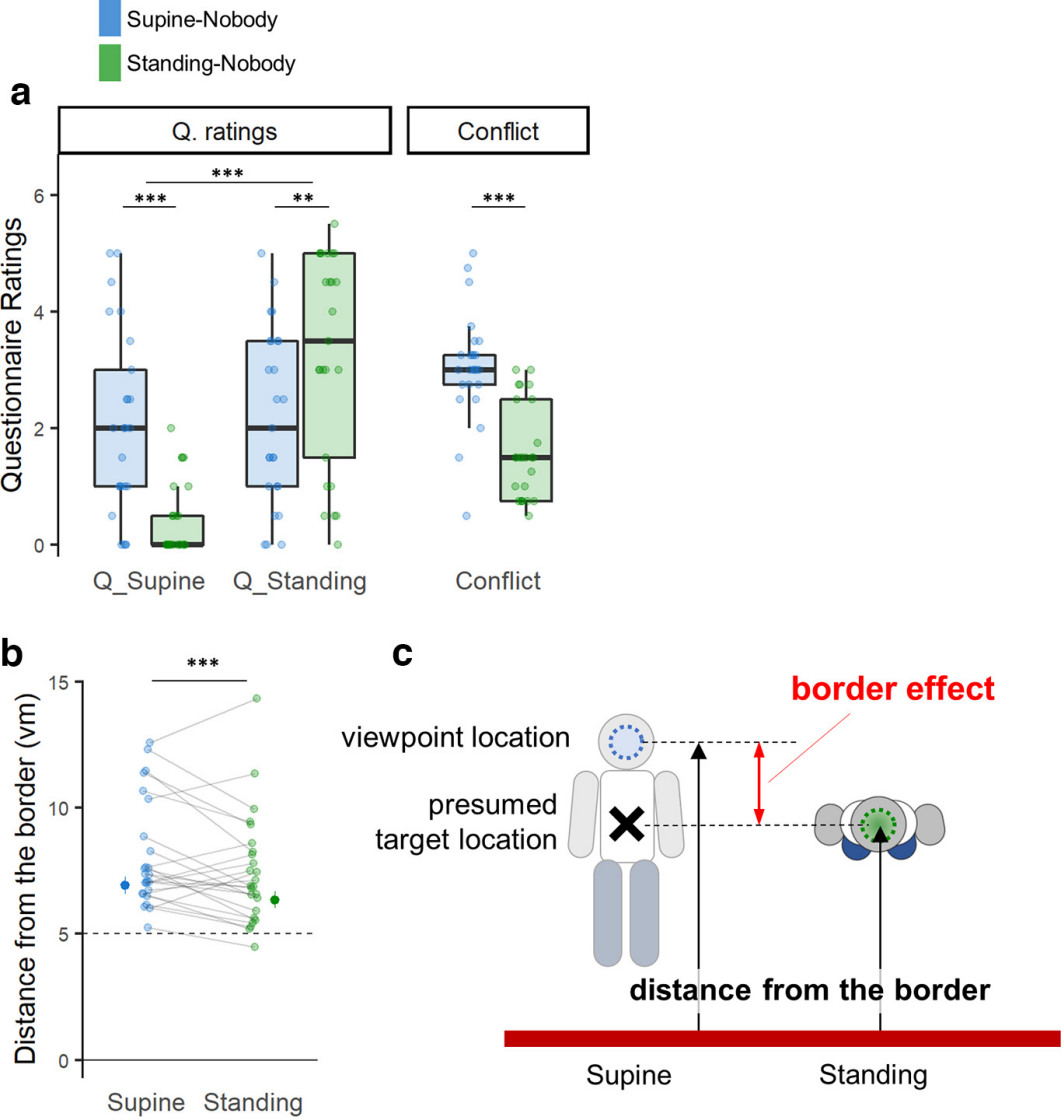
Effect of physical BO on experienced BO and on navigational behavior in VR. ***a***, When participants were physically supine (blue), they had a stronger experience of being supine in VR while they felt less as if they were standing, compared with when they were actually standing upright. The conflict score indicates how much their experienced BO in VR conflicted with their physical BO. The results showed that the conflicts were significantly larger in the physically supine BO condition than in the standing BO condition. Also, overall, when compared, regardless of their physical BO, participants reported a significantly stronger experience of being standing in VR, suggesting the standing BO as a presumed BO in VR. ***b***, Participants’ reached location was farther away from the border when they were physically supine compared with when they were standing upright. ***c***, A top-view schematic figure depicting the border effect. The changes in the distance from the border possibly reflected changes in self-location (or body boundaries) that are related to the change in the experienced BO in VR. Consequently, the distance from the border (calculated with respect to the viewpoint location) was greater when they were physically supine compared with when they were standing upright. **0.001 ≤ *p* < 0.01, ***p < 0.001.

To assess the impact of physical BO on the experienced BO and navigational behavior in VR, we compared virtual navigation when our participants were supine versus when they were standing without any avatar shown (Nobody conditions; Fig. 1*a*, blue and green; Table 2). As predicted, we found a significant influence of physical BO on the experienced BO during the virtual navigation task in VR, as assessed through subjective questionnaire ratings and implicit behavioral measures. We found a significant effect of physical BO on questionnaire ratings pertaining to the experienced BO in VR (Fig. 2*a*, left). Our participants reported significantly higher Q_Supine ratings (experience of being supine in VR; *r* = 0.783, *p* < 0.001) and lower Q_Standing ratings (experience of standing upright in VR; *r* = 0.593, *p* = 3.01e-03) when their physical BO was supine (i.e., Supine-Nobody condition) than when standing (i.e., Standing-Nobody condition). These results indicate that experienced BO in VR is influenced by BO of the physical body. Therefore, when participants’ physical BO was standing upright (i.e., Standing-Nobody), they felt as if they were standing in VR, rather than being supine (Q_Standing > Q_Supine; *r* = 0.841, *p* < 0.001). However, in the Supine-Nobody condition, Q_Supine and Q_Standing had equal ratings (i.e., did not differ; *r* = 0.093, *p* = 0.64), revealing an ambiguity in our participants’ experienced BO when their physical BO was supine and when they did not receive additional visual cues regarding BO in the virtual environment (i.e., Nobody condition). In addition, we found overall higher Q_Standing ratings compared with the Q_Supine (*r* = 0.566, *p* < 0.001), suggesting that our participants preferably experienced a standing BO in VR. The conflict score (see Materials and Methods) confirmed these findings, revealing higher conflict scores when participants were physically supine versus upright (Fig. 2*a*, right; *r* = 0.762, *p* < 0.001), possibly reflecting an incongruence between the participants’ physical BO (supine) and their preferably experienced BO in VR (upright).

**Table 2:** Statistical table

Figure	Variables	Data structure	Type of test	Power
(a) Figure 2*a*	Q_Supine: supine-nobody vs standing-nobody	Ordinal data	Wilcoxon signed-rank test	*r* = 0.783
(b) Figure 2*a*	Q_Standing: supine-nobody vs standing-nobody	Ordinal data	Wilcoxon signed-rank test	*r* = 0.593
(c) Figure 2*a*	Q_Standing vs Q_Supine in standing-nobody	Ordinal data	Wilcoxon signed-rank test	*r* = 0.841
(d) Figure 2*a*	Q_Standing vs Q_Supine in supine-nobody	Ordinal data	Wilcoxon signed-rank test	*r* = 0.093
(e) Figure 2*a*	Conflict score: supine-nobody vs standing-nobody	Ordinal data	Wilcoxon signed-rank test	*r* = 0.762
(f) Figure 2*b*	Distance from the border: supine-nobody vs standing-nobody	γ distribution	Mixed effects model (fixed effect: BO, random effect: Subject, random slope: BO, nested: object)	df = 1, *F* = 11.08
(g) Figure 3*a*	Q_Supine: supine-nobody vs supine-body	Ordinal data	Wilcoxon signed-rank test	*r* = 0.720
(h) Figure 3*a*	Q_Standing: supine-nobody vs supine-body	Ordinal data	Wilcoxon singed-rank test	*r* = 0.595
(i) Figure 3*a*	Q_Standing vs Q_Supine in supine-body	Ordinal data	Wilcoxon signed-rank test	*r* = 0.759
(j) Figure 3*a*	Conflict score: supine-nobody vs supine-body	Ordinal data	Wilcoxon signed-rank test	*r* = 0.800
(k) Figure 3*b*	Distance from the border: supine-nobody vs supine-body	γ distribution	Mixed effects model (fixed effect: BO, random effect: Subject, random slope: BO, nested: object)	df = 1, *F* = 16.56
(l) Figure 4*a*	Distance from the border	γ distribution	Mixed effects model (fixed effect: BO * avatar, random effect: Subject, nested: object)	BO: df = 1, *F* = 10.51; avatar: df = 1; *F* = 44.65; interaction: *F* = 0.51
(m) Figure 4*b*	Distance error	γ distribution	Mixed effects model (fixed effect: BO * avatar, random effect: Subject, nested: object)	BO: df = 1, *F* = 0.57; avatar: df = 1; *F* = 7.78, interaction: *F* = 0.66
(n) Figure 4*c*	Trial time	Log-normal distribution	Mixed effects model (fixed effect: BO * avatar, random effect: Subject, nested: object)	BO: df = 1, *F* = 32.9; avatar: df = 1; *F* = 0.01, interaction: *F* = 0.09
(o) Figure 4*d*	Navigated distance	Log-normal distribution	Mixed effects model (fixed effect: BO * avatar, random effect: Subject, nested: object)	BO: df = 1, *F* = 0.06; avatar: df = 1, *F* = 25.9; interaction: *F* = 0.02

10.1523/ENEURO.0218-23.2023.f2-1 Extended Data Figure 2-1 Distance from the border serves as an objective behavioral measure of experienced BO (i.e., supine position) in VR. a, Distance from the border data were significantly correlated with ratings of Q_Supine (df = 1, F = 19.08, p < 0.001, n = 25). A mixed-effect model was used to assess their relationship. b, Their relationship at the within-subject level was further assessed through in-depth analysis. The distance from the border and Q_supine data were re-calculated and plotted with respect to the Supine-Nobody condition (i.e., scanner condition). We found that a change in the distance from the border of a subject in a condition was significantly associated with the change in the Q_supine rating of the subject in the condition. **: 0.001 <= p < 0.01, ***: p < 0.001. Download Figure 2-1, TIF file.

The significant influence of the physical BO on the experienced BO was further validated by an implicit navigation behavior during the task: the distance from the border when participants stopped at the end of each retrieval trial. Our participants stopped at a significantly larger distance from the border in Supine-Nobody condition compared with Standing-Nobody condition (mixed-effect regression; *d* = 0.034, predicted_difference = 0.58 virtual meter (vm), df = 1, *F* = 11.08, *p* < 0.001, *n* = 25; Fig. 2*b*), as if they wanted to avoid that their legs would hit the border of the arena. We note that every task object was placed at equal distance (5 vm away) from the border and that participants were approaching the targets facing the border (majority of trials, 98.1%). Accordingly, we argue that the border effect (i.e., the larger/smaller distance from the border) reflects an implicit incorporation of the participant’s physical BO into navigation behavior (Alsmith and Longo, 2014; Moon et al., 2022). Thus, even when not seeing a virtual agent during virtual navigation (as in Moon et al., 2022), supine participants stopped farther from the border, behaviorally corroborating the subjectively experienced supine BO (Fig. 2*c*). Further analysis corroborated this association by showing that the distance from the border was significantly correlated with the strength of the participant’s subjective experience of being supine (Q_Supine; df = 1, *F* = 19.08, *p* < 0.001, *n* = 25; Extended Data Fig. 2-1).

In addition, we also investigated the impact of the physical BO on other navigation performance measures (i.e., distance error, navigated distance, trial time). The impact of BO on these was assessed together with the effects with body view (also to check their possible interactions) and will be reported in a separate section (see the last section of the Results).

### Effect of the virtual body on experienced BO and conflicts

**Figure 3.**
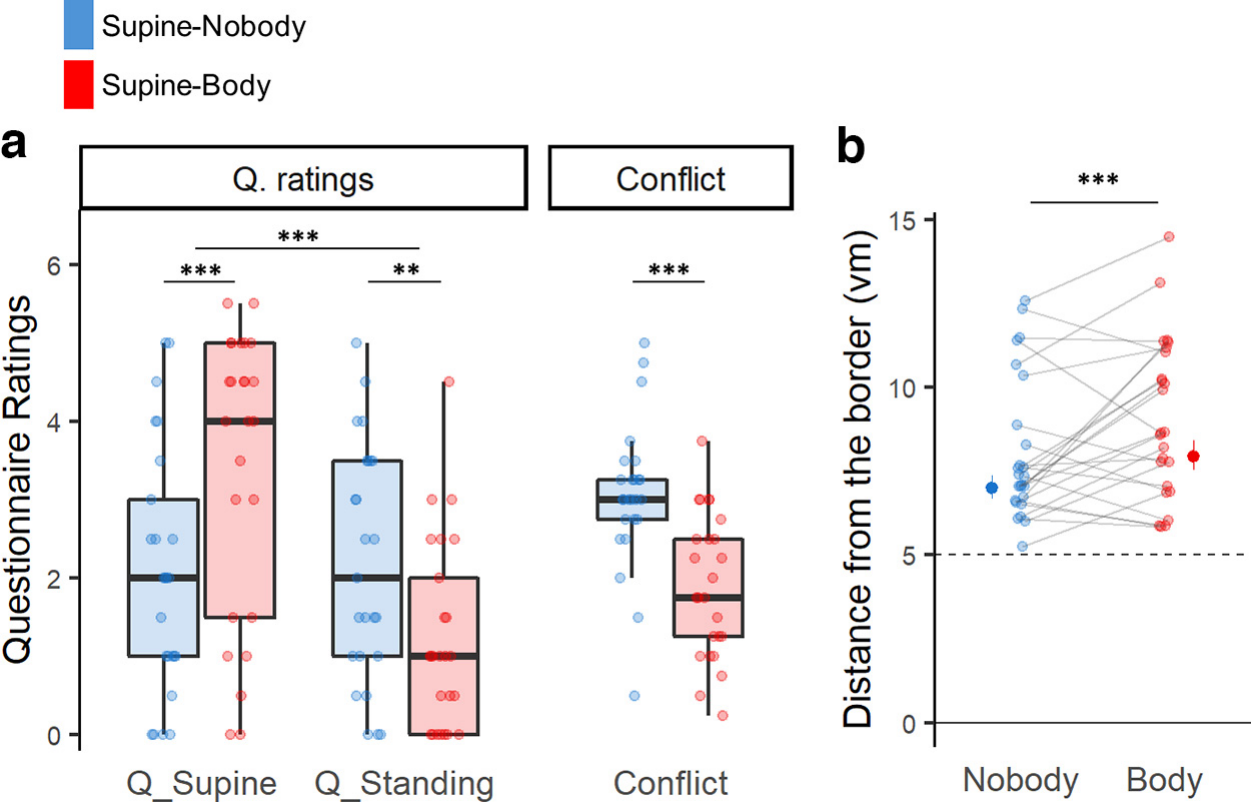
Experienced BO affected by the BO-congruent avatar. ***a***, When the participants were physically supine, presenting an avatar with supine posture (i.e., Body condition) strengthened the subjective experience of being supine and reduced the experience of standing upright compared with the Nobody condition, where no avatar was shown. The conflict score confirms that showing the BO-congruent avatar significantly reduced the conflict of the BO. When compared, regardless of the presence of the avatar, they felt more as if they were supine than standing, again highlighting the impact of the physical BO on the experienced BO in VR (Q_supine > Q_standing, overall). ***b***, We also reproduced previous findings of a significant shift in the distance from the border between body and nobody conditions, suggesting that such drifts in self-location are associated with changes in the experienced BO in VR. **0.001 ≤ *p* < 0.01, ***p < 0.001.

We next analyzed the impact of the view of the BO-congruent avatar on experienced BO in VR. For this, we analyzed the two experimental conditions in which our participants were physically supine, comparing the condition showing the virtual scene without any avatar (i.e., Supine-Nobody condition) with the condition presenting the virtual scene and the supine virtual avatar (i.e., Supine-Body condition). In the latter condition, the posture of the avatar was congruent with the participants' physical posture (supine). Assessing whether an avatar with a congruent BO (with respect to the participant’s physical BO) affects the experienced BO in VR, we show that when participants were physically supine and presented with an avatar (i.e., Supine-Body condition), they have an enhanced experience of being supine (Fig. 3*a*, left; Q_Supine; *r* = 0.720, *p* < 0.001, *n* = 25) and a reduced experience of standing upright (Q_Standing; *r* = 0.595, *p* = 2.95e-03), compared with the condition without the avatar (i.e., Supine-Nobody condition). Furthermore, in Supine-Body condition, the ratings for Q_Supine were significantly higher than those for Q_Standing (Fig. 3*a*, left; *r* = 0.759, *p* < 0.001), which was not the case in Supine-Nobody condition (*r* = 0.093, *p* = 0.64). The data, hence, demonstrate reduced ambiguity in experienced BO when the avatar was present. This is further confirmed by a lower conflict score in the condition with a body-congruent avatar compared with the condition with no avatar (Fig. 3*a*, right; *r* = 0.800, *p* < 0.001).

In addition, congruently with the questionnaire results, we found an even larger border effect (drift in self-location), with participants keeping larger distances from the border, in Supine-Body condition compared with Supine-Nobody condition (mixed-effect regression; *d* = 0.045, predicted_difference = 0.95 vm, df = 1, *F* = 16.56, *p* < 0.001, *n* = 25; Fig. 3*b*), confirming previous data by Moon et al. (2022). The association between the border effect and the experienced BO was confirmed by the significant correlation between the border distance and Q_Supine rating (df = 1, *F* = 19.08, *p* < 0.001, *n* = 25; Extended Data Fig. 2-1).

### Effect of physical BO and 1PP avatar on the navigation performances

**Figure 4.**
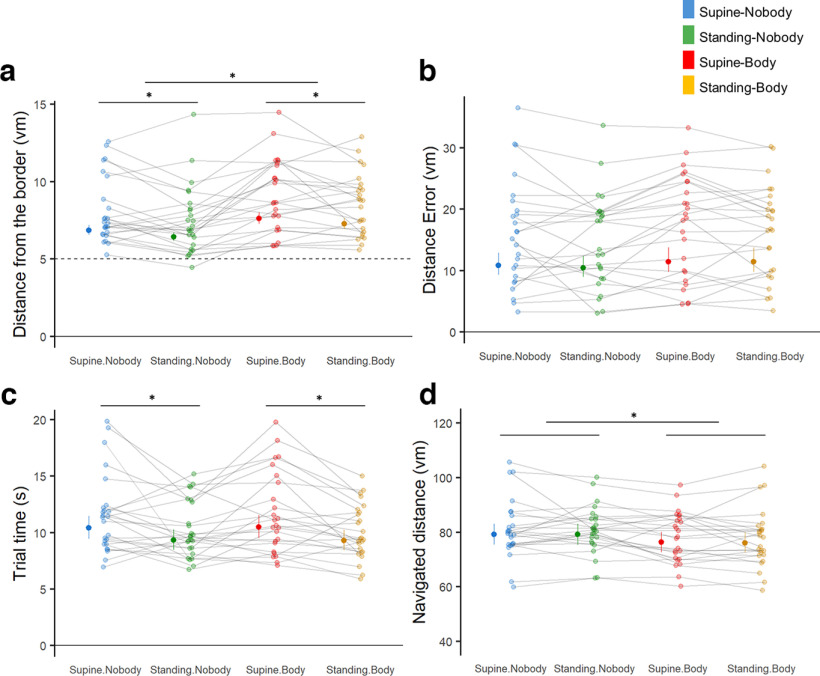
Physical BO and the presence of the avatar affected navigation behaviors in VR. ***a***, Distance from the border, which arguably reflects experienced BO in VR, was significantly larger when participants were physically supine rather than standing, and also when a supine avatar was presented in a first-person viewpoint position than no avatar was shown. Predicted mean value per condition in order: 6.86, 6.43, 7.62, and 7.27 vm. ***b***, Distance Error, indexing spatial navigation precision, did not significantly differ among the four conditions. Predicted mean value per condition in order: 11.06, 10.60, 11.69, and 11.69 vm. ***c***, Participants spent significantly less time in the retrieval phase when they were physically standing compared with when they were physically supine, regardless of the presence of the avatar. Predicted mean value per condition in order: 10.40, 9.34, 10.48, and 9.29 s. ***d***, The navigated distances were significantly reduced in the Body conditions (i.e., Supine-Body and Standing-Body) compared with the Nobody conditions (i.e., Supine-Nobody and Standing-Nobody). Predicted mean value per condition in order: 79.18, 79.12, 76.29, and 76.07 vm. No significant interactions were found for all four parameters. A dedicated mixed-effects model was used for statistical assessments of each parameter. Single data points represent the measure per participant, and dot and whiskers on the left of each data cloud indicate predicted mean value and 95th percentile, respectively. *p < 0.05.

Based on the data from our four experimental conditions, we assessed the impact of both physical BO and presenting a supine virtual avatar on navigation performance. Either of those effects and their possible interaction were simultaneously taken into account through a dedicated mixed-effect model for each behavioral parameter. Through these analyses, we found a significant impact of physical BO on trial time: the time participants spent per retrieval trial was significantly shorter when they were physically standing upright versus supine (mixed-effect regression; *d* = 0.36, predicted_difference = 1.13 s, df = 1, *F* = 32. 90, *p* < 0.001, *n* = 25; Fig. 4*c*). However, we did not observe any significant influence of physical BO on spatial memory accuracy (i.e., distance error; *d* < 0.01, *F* = 0.57) and navigated distance per trial (*d* = 0.01, *F* = 0.06). On the other hand, seeing a virtual agent while navigating (i.e., Body condition) significantly reduced navigated distance compared with the conditions without an avatar (mixed-effect regression; *d* = 0.22, predicted_difference = 2.97 vm, df = 1, *F* = 25. 95, *p* < 0.001; Fig. 4*d*), while it did not affect the other navigation performance measures (i.e., distance error: *d* < 0.01, *F* = 7.78; trial time: *d* < 0.01, *F* < 0.01). This suggests more efficient navigation (i.e., less distance traveled to reach the same location) in the avatar condition. Notably, we did not find significant interactions between physical BO and avatar conditions on any of the navigation performance measures. Overall, these data suggest that the supine physical BO (i.e., BO in MRI) had a significant negative impact on virtual navigation (i.e., trial time) and that seeing a virtual agent can affect some aspect of spatial navigation behavior in VR (i.e., navigated distance per trial).

## Discussion

In this study, we demonstrate that physical BO (standing or supine participants) modulates experienced BO and some navigational measures in VR. Our data also show that a standing BO is preferred during spatial navigation, because participants were more likely to experience a standing BO than a supine BO and because they were faster in retrieving locations when they were standing upright (i.e., shorter navigation time). We also corroborate previous reports by demonstrating that showing a virtual avatar in a supine position reduces the conflict between supine physical BO and preferred BO (i.e., standing) and improves an aspect of spatial navigation (i.e., shorter navigation path). Because many navigation-related neuroimaging studies are done in the MRI scanner (Doeller et al., 2010; Kunz et al., 2015; Horner et al., 2016; Stangl et al., 2018; Bierbrauer et al., 2020; Moon et al., 2022), where physical BO is constrained to be supine (Taube et al., 2013; Steel et al., 2021), our study provides important information about experienced BO and potential biases arising from it, which should be considered when spatial navigation studies in the scanner are designed or interpreted.

First, we demonstrated that participants’ physical BO modulated their experienced BO in VR as measured by subjective questionnaire ratings and by the border effect. Concerning BO ratings, we found that when participants navigated the virtual environment without seeing an avatar, their experienced BO was influenced by their physical BO: standing BO ratings increased and supine BO ratings decreased when they were physically standing upright, and vice versa. These subjective data were corroborated by the border effect in navigation behavior (i.e., the distance from the border of the navigation arena during retrieval trials). When navigating to the target location, our participants kept a larger distance from the border while their physical body was in supine versus standing BO (Fig. 2*b*,*c*). We note that both conditions were visually identical (no avatar in either condition) and differed only in physical BO. Thus, the border effect cannot result from visual differences between conditions, but rather by the different physical BO, associated with differences in experienced BO: the space occupied by the body expands forward in the supine BO (but see Alsmith and Longo, 2014; Moon et al., 2022). The finding of a positive correlation between the distance from the border with the strength of the participant’s subjective experience of being supine confirms this interpretation (Extended Data Fig. 2-1). Accordingly, we argue that the border effect is an implicit change in navigational behavior when participants are in supine physical BO versus standing. Importantly, this navigational parameter provides an objective and repeated proxy of one’s experienced BO, eliminating potential biases that may arise from explicit questionnaires. This effect is of direct relevance for spatial navigation studies performed during fMRI (Doeller et al., 2010; Kunz et al., 2015; Stangl et al., 2018; Bierbrauer et al., 2020) where supine BO is unavoidable. Apart from altering some aspects of navigation behavior (as shown by the border effect), the supine BO may also affect place/grid cell-related brain activity (i.e., grid cell-like representation in entorhinal cortex; Doeller et al., 2010; Jacobs et al., 2013; Nadasdy et al., 2017; Maidenbaum et al., 2018; Moon et al., 2022) or the activation of other regions involved in spatial navigation such as retrosplenial cortex (Vann et al., 2009; Mitchell et al., 2018; Bierbrauer et al., 2020; Alexander et al., 2023). For example, the border effect strongly suggests that the reference point to which a reference frame in VR is anchored was shifted, and this could possibly lead to a corresponding shift of place/grid field map encoding the location of a virtual agent in VR. Furthermore, BO of one’s physical body could also affect the orientation of the reference frame anchored to it. For instance, “heading forward” may mean navigation to the ceiling when one is supine in MRI, rather than navigation on the horizontal plane as in the virtual arena. Thus, we believe that most of the human virtual navigation studies describe neural activities from the reference frame anchored to the virtual agent in VR, not the physical body. However, these two distinct reference frames (possibly encoded by distinct cognitive maps in MTL) may conflict in some experimental conditions (e.g., Body condition), and supine physical BO might contribute to the conflict. The influence of physical BO on the experienced BO suggest that a virtual agent (even when it is invisible in Nobody condition) and the participant’s body are functionally linked to each other during virtual navigation. The association could be mediated by the intrinsic signals from the physical body (e.g., vestibular and proprioceptive signals; Pfeiffer et al., 2014; Lenggenhager and Lopez, 2015; Park and Blanke, 2019). Thus, direct changes or disruptions to the bodily inputs could influence both the navigation experience (BO) and related navigation behaviors (i.e., border effect). This hypothesis needs further investigation in future studies using experimental manipulation of those bodily signals, such as electrical vestibular stimulation (Sluydts et al., 2020).

Second, our data show that standing is the preferred experienced BO during virtual navigation. We found that a conflict between the physical BO and the experienced BO in VR (as measured by the conflict score) was larger when our participants were physically supine versus standing upright. Thus, when they were physically standing upright, they felt as if they also were standing in the virtual arena during navigation (supported by bodily signals). In contrast, when they were in supine BO in the real world, their reported experience of BO in the virtual arena was more ambiguous (between supine and standing). Considering the influence of the supine physical BO that we discussed above, the observed ambiguity could be the result of incongruence between the preferred BO in VR (i.e., standing upright) and the supine physical BO (arguably, mediated by vestibular and proprioceptive cues from the supine body). We argue that the preferred standing BO during virtual navigation most likely originates from our daily experience of upright position during physical navigation. Human brain mechanisms of spatial navigation could have adapted to the evolutionary change in BO and been optimized for navigation in standing upright BO (also based on body structure, sensory system, and lifestyle distinguished from other animals; Ekstrom, 2015). This was also indirectly supported by the influences of the physical BO and experienced BO on spatial navigation performance in VR. We observed a decrease in time to retrieve and navigate to the target when participants’ physical BO was standing (Fig. 4*a*), suggesting that standing BO (i.e., preferred BO during navigation as suggested above) facilitates some spatial navigation processes. Alternatively, this effect could also be linked to changes in space perception (e.g., visual vertical judgment; which is possibly related to the prediction of the destination ahead of straight navigation) that have been reported to be better in the upright position (compared with the supine position), by the contribution of vestibular gravitational signals (Lopez et al., 2008; Lopez and Blanke, 2010). Size or distance perceptions have been reported to be better while upright than supine: supine BO makes the size or the distance more underestimated (Kim et al., 2022). Alternatively, although the VR scene presented in the HMD was the same, our participants might have experienced it from an elevated perspective (i.e., as if they were looking down; which is a more likely situation while standing than while supine) when they were physically upright but not when they were supine. An elevated perspective has been associated with faster response times in visuospatial tasks (vs eye-level or lowered perspective; Schwabe et al., 2009). Indeed, a previous study by Ionta et al. (2011) reported that BO in a virtual space experienced by participants in the MRI scanner (i.e., looking-up vs looking-down) could be altered by multisensory bodily signals while keeping the visual input constant. Altogether, the present findings suggest that the human navigation system “has a preference” for navigation in the upright BO, and that—although virtual navigation can simulate natural navigation (as if upright) with some extent of ambiguity—it may nonetheless impact some aspects of navigation in supine BO (as in MRI). It is possible that the fixed visual angle of 15° in our experiment (adjusted to be more upward than usual, but still closer to the case of upright) could affect the preference for the upright BO and the better performance in the same condition. While this may compromise the generality of our findings, our results are still very important as the majority of virtual navigation research uses a visual angle that is almost horizontal.

Finally, we show that also the presentation of a supine avatar during virtual navigation influences both experienced BO and navigation behaviors in VR. Concerning experienced BO in VR, we report that the avatar in the supine position was associated with a stronger sensation of being in supine BO in VR. This finding is compatible with an important role of bodily multisensory cues in virtual navigation experience. It should be stressed that this rather simple experimental manipulation (i.e., showing a supine avatar during virtual navigation) significantly reduced the conflict between the experienced BO and the physical BO when participants were lying supine (which is the adopted posture in fMRI acquisitions), as highlighted by the lower conflict score in Supine-Body condition compared with Supine-Nobody condition. As discussed above, participants’ experienced BO in the Supine-Nobody condition (i.e., the typical condition in MRI) is experienced as ambiguous, arguably because of the incongruency between preferred BO and the influence of supine physical BO. However, our results suggest that the addition of a seen avatar reduces this ambiguity and the related sensory-experiential conflict (i.e., conflict score). Importantly, we show that these subjective BO changes were reflected in the border effect, reproducing previous results in the MRI scanner (Moon et al., 2022). Of note, this border effect, as induced by a supine avatar (Supine-Body condition), further increased the distance from the border compared with Supine-Nobody condition, where we observed the border effect induced by supine physical BO when compared with Standing-Nobody. Thus, the border effect further increased when the subjective experience of being supine in VR was enhanced by (1) supine physical BO and again by (2) the presence of a supine avatar (Standing-Nobody < Supine-Nobody < Supine-Body). This finding was further supported by the significant correlation between the border effects and Q_supine ratings, and vice versa (Extended Data Fig. 2-1). Moreover, seeing a supine avatar also reduced the navigated distance per trial without increasing retrieval errors, suggesting improved spatial navigation in the conditions with an avatar (Fig. 4*b*) as was the case in the similar study in MRI (Moon et al., 2022). This is again of relevance to human navigation studies using virtual navigation paradigms, and in particular to those paradigms using fMRI (Taube et al., 2013; Steel et al., 2021). Overall, our data suggest that body-related cues (e.g., view of a body posture-congruent avatar) as well as signals from the body systematically evoke conflicts to different degrees depending on the actual conditions; this knowledge should be used to improve and better understand experienced BO and navigation behaviors in virtual navigation, especially when a supine BO is necessary as in the fMRI studies (Doeller et al., 2008, 2010; Taube et al., 2013; Stangl et al., 2018; Bierbrauer et al., 2020; Moon et al., 2022).

Notably, we elucidate the limitations stemming from the presentation of a supine avatar, despite its merits we have shown above. The introduction of a supine avatar may influence a wide range of neural activities from the rudimentary level visual perception to higher-level cognitions related to sense of self and spatial navigation, as already reported in the previous studies (Iriye and Ehrsson, 2022; Moon et al., 2022, 2023). Therefore, it is essential to properly consider these aspects before adopting the supine avatar in experimental design.

Studies on imagined navigation (Bellmund et al., 2016; Horner et al., 2016), in which participants’ imagined BO was not restricted, showed similar neural correlates to virtual navigation. One might argue that these findings suggest a limited influence of supine BO in the scanner on the human navigation system. However, similar neural correlates between the imagined navigation and virtual navigation might also indicate that virtual navigation without any physical displacement is more similar to “imagined navigation” than to “upright real-world navigation.” Thus, without direct comparison between the neural correlates of supine virtual navigation and those of physical spatial navigation, we cannot really conclude that there is no major influence of the supine BO on the neural activity in the human brain.

Collectively, our results highlight the importance of the physical BO and the relevant visual cue (i.e., BO-indicating body view) on virtual navigation by showing their influence on both subjectively experienced BO and the navigation behaviors in VR. Through this study, we confirm that standing BO is preferred during navigation with solid evidence, which has been considered so far without much evidence. By comparing the conventional condition in MRI with others (including the preferred condition) across a range of aspects, our data provide a more fine-grained understanding of the potential, but often overlooked, effects that could be caused by the constraint in MRI. Importantly, the present data show that the addition of a seen avatar can be a simple but powerful method to resolve the ambiguity of the experience of BO in MRI and possibly to improve some navigation performance (i.e., Moon et al., 2022). Our results strongly suggest that multisensory body-related aspects should also be cautiously considered in the experimental design of any virtual navigation study. Finally, we propose the border effect as a robust measure that allows assessing the potential biases and confounds in experienced BO during virtual navigation, and thereby helps control them.

## Synthesis

Reviewing Editor: Ifat Levy, Yale School of Medicine Decisions are customarily a result of the Reviewing Editor and the peer reviewers coming together and discussing their recommendations until a consensus is reached. When revisions are invited, a fact-based synthesis statement explaining their decision and outlining what is needed to prepare a revision will be listed below. The following reviewer(s) agreed to reveal their identity: Roberto Bottini.

The authors adequately revised in response to most of the reviewers’ comments.

Please expand the discussion of potential limitations of adding an avatar, such as the potentially altered visual experiences and their effects.
