# Supplementary figures and images for: Physical Body Orientation Impacts Virtual Navigation Experience and Performance
Source: eNeuro. 2023 Nov 23;10(11):ENEURO.0218-23.2023. doi: 10.1523/ENEURO.0218-23.2023 (PMC10683533; doi:10.1523/ENEURO.0218-23.2023)

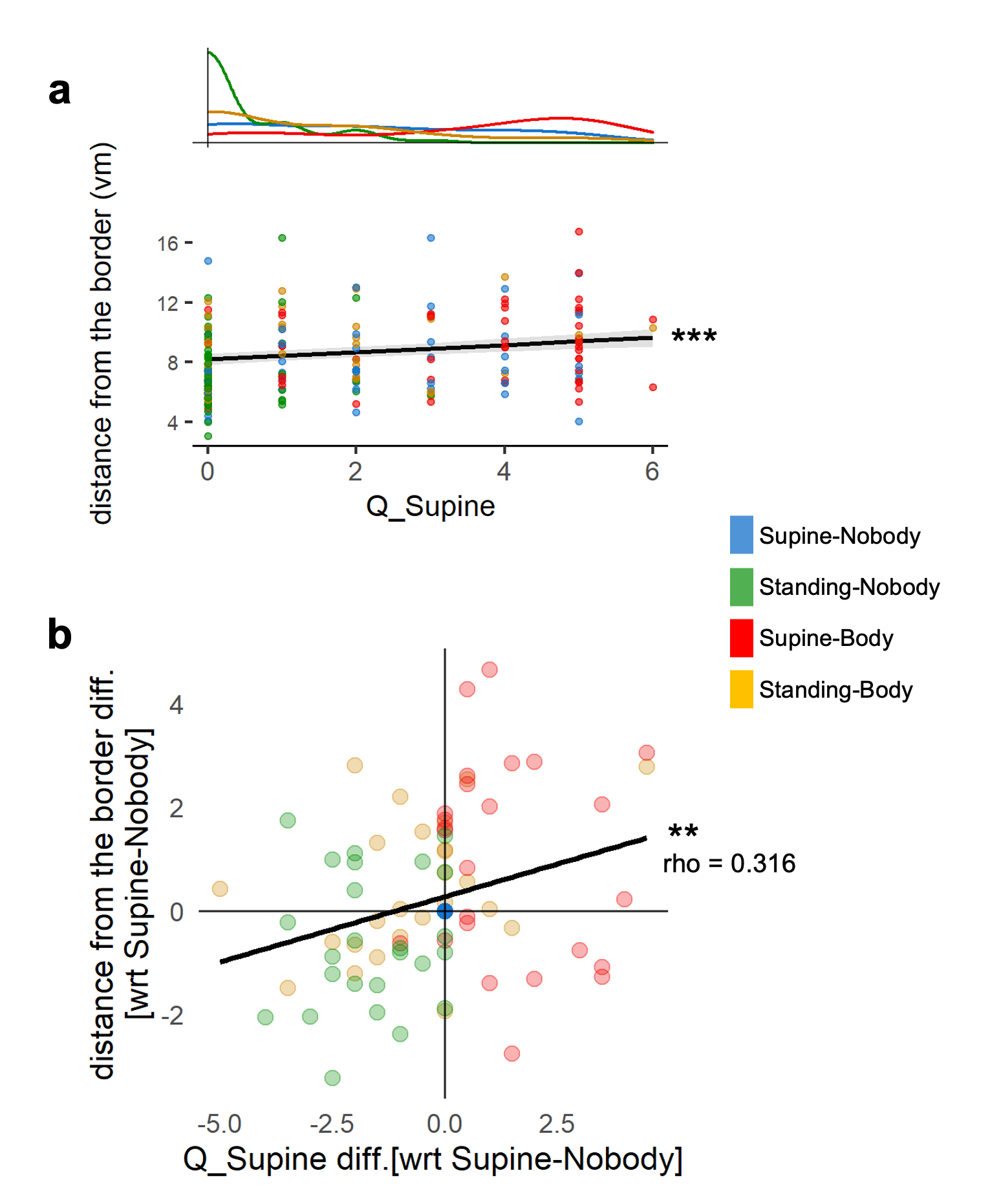

Supplement: Extended Data Figure 2-1 — Distance from the border serves as an objective behavioral measure of experienced BO (i.e., supine position) in VR. a, Distance from the border data were significantly correlated with ratings of Q_Supine (df = 1, F = 19.08, p < 0.001, n = 25). A mixed-effect model was used to assess their relationship. b, Their relationship at the within-subject level was further assessed through in-depth analysis. The distance from the border and Q_supine data were re-calculated and plotted with respect to the Supine-Nobody condition (i.e., scanner condition). We found that a change in the distance from the border of a subject in a condition was significantly associated with the change in the Q_supine rating of the subject in the condition. **: 0.001 <= p < 0.01, ***: p < 0.001. Download Figure 2-1, TIF file. [file enu-eN-NWR-0218-23-s03.tif]
